# The Determinants of Sexual Intercourse Before Age 16 Years Among Rural Jamaican Adolescents

**DOI:** 10.1100/tsw.2007.94

**Published:** 2007-04-09

**Authors:** Olaniyi J. Ekundayo, Joana Dodson-Stallworth, Michele Roofe, Inmaculada B. Aban, Laura H. Bachmann, Mirjam C. Kempf, John Ehiri, Pauline E. Jolly

**Affiliations:** ^1^ Department of Epidemiology, School of Public Health, University of Alabama at Birmingham, Birmingham, AL, USA; ^2^ Division of HIV/AIDS Prevention, National Center for HIV, STD, and TB Prevention Centers for Disease Control and Prevention, Atlanta, GA, USA; ^3^ North East Regional Health Authority Ocho Rios, St Ann, Jamaica; ^4^ Department of Biostatistics, School of Public Health, University of Alabama at Birmingham, Birmingham, USA; ^5^ School of Medicine, Division of Infectious Diseases and School of Public Health, Department of Epidemiology, University of Alabama at Birmingham, Birmingham, USA; ^6^ Department of Maternal and Child Health, School of Public Health, University of Alabama at Birmingham, Birmingham, USA

**Keywords:** adolescents, early sexual debut

## Abstract

Individual and family factors have been hypothesized to influence adolescent sexual behavior, but the extent to which this is true for adolescents in Jamaica as a whole and for those in rural areas in particular, has not been well studied. The objective of this study was to identify individual and family factors associated with initiation of sexual activity before the age of 16 among rural adolescents in Jamaica. We analyzed data for 469 sexually experienced adolescents attending public high schools in the rural parish of Hanover. Multivariate logistic regression was used to predict independent influences of these factors. The mean age at sexual debut was 11 years for boys and 15 years for girls. Early adolescent sexual activity was associated with liberal attitudes about negative sexual outcomes (OR = 1.96, 95%CI = 1.34-2.87) and first sexual partner not being a steady boyfriend or girlfriend (OR = 4.19, 95%CI = 1.62-10.84). Female gender (OR = 0.16, 95%CI = 0.07-0.36) and older age at time of survey were protective (OR = 0.40, 95%CI = 0.32-0.52). Girls who were early starters were more likely to have been initiated by partners who were not steady boyfriends. They also reported liberal attitude towards negative sexual outcomes. Boys were mainly influenced by liberal attitude towards negative sexual outcomes. Being older was protective for both genders. Considering the high rates of HIV and adolescent pregnancy in this population, reproductive health programs that attempt to delay age at first sex should begin early in primary school before adolescents become sexually active.

## INTRODUCTION

In the past three decades, there has been a considerable decline in the age at which adolescents initiate first sexual intercourse, both in the developed and developing countries[1,2]. Early initiation of sex exposes teenagers to the risk of unintended pregnancy[3] and sexually transmitted infections (STIs), including Human Immunodeficiency Virus (HIV)[3]. Adolescent child bearing has been linked to higher rates of stillbirth[4], maternal and child morbidity and mortality, limited life opportunities, and lower socio-economic status[5]. Research efforts in recent years, especially in developed countries, have been directed towards identifying factors associated with early adolescent sexual activity and mounting programmatic strategies to modify sexual behavior.

Globally, early adolescent sexual activity and teenage pregnancy remains a recurring public health issue. In Jamaica for instance, available data, indicates that adolescents initiate sexual activity early and engage in risky sexual behavior. In the 2001 Adolescent Condom Survey, 63% of females and 83% of males (10-19 years old) reported having had sexual intercourse[6]. In another study, adolescent males reported earlier onset of sexual activity than females, mean age at first intercourse was as low as 9.4 years for boys and 11.3 years for girls[2]. In the Adolescent Condom Survey, only 42% of respondent 15-19 years old and 26% of 10-14 years old used contraceptives the first time they had sexual intercourse[6]. Consequently, the pregnancy rate among Jamaican adolescents (79 live births per 1,000) although declining, compared to 112 per thousand in 1997, still represents one of the highest in the Caribbean[7]. In fact adolescent pregnancy in this country has been described as a social and public health problem[8]. Research findings indicate that before they reach the age of 20 years, 40% of Jamaican women have been pregnant at least once, and over 80% of these adolescent births are mistimed or unwanted[9]. Adolescent pregnancy is pervasive throughout the country, both in the urban and rural areas. In Hanover, the most rural parish in Jamaica, approximately 25% of the births in 1997 were to teen mothers[10].

Apart from the health consequences of teenage childbearing, the social consequences are also enormous for the Jamaican adolescent mother. They are often limited economically, usually abandoned by partners, and frequently unable to complete school. For instance, among ever-pregnant adolescent females in the country, almost one-third became pregnant while still in school, and only 16% of these returned to school after the birth of their child[9]. The majority of teenage pregnancies are often unintended and as a result, many teenagers are often desperate and willing to risk their life to terminate them[9]. In Jamaica, abortion is only legal for medical reasons and therefore pregnant adolescents often seek illegal abortions from untrained and unqualified personnel who conduct abortions under unsafe conditions. The resulting infections and complications have been reported as an important cause of maternal morbidity and mortality in the country[11].

Finally, early teenage sexual activity is associated with a higher likelihood of subsequent unprotected sex and multiple sexual partners, putting adolescents at risk for STIs including HIV. Most AIDS cases in Jamaica occur among young people ages 20 to 39, meaning that most people are infected with HIV as adolescents and young adults[12]. In 2004, HIV was the second leading cause of death for both young men and women in Jamaica (age group 15-24 years old)[12]. The small, rural parish of Hanover has the third highest rate (217.1/100,000 population) of HIV infection in the country[13].

In Jamaica, the only available study conducted to identify predictors of early sexual intercourse was based on retrospective information from women aged 15 to 50 years, with potential limitation of recall bias[14]. Most information has been derived largely from qualitative research and anecdotal evidence[9,15]. No empirical research has been conducted to quantitatively identify correlates of early sexual debut among Jamaican adolescents who had recently experienced intercourse particularly in a rural setting. Findings from qualitative studies confirmed that a combination of individual and family level factors is associated with early sexual activity. Early coitus for girls has been reported to be associated with sex in exchange for money to meet economic needs, exploitation by male relatives, sexual curiosity, seeking fun and pleasure, love and affection, pressure (from boys, peers and adult men), force and physical violence, desire to have a baby, and lack of parental monitoring[9,15]. On the other hand, boys were mostly motivated by pleasure, wanting to have a baby, peer pressure and economic gains from older females[9,15].

In this study, our goal was to identify factors predicting early initiation of sexual activity among high school adolescents in the rural parish of Hanover, Jamaica, a parish with high rates of adolescent pregnancy and STIs. We compared those who had sexual experience before the age of 16 -the age of legal consent in Jamaica[16] with those who initiated sexual intercourse at age 16 and older. We hypothesize that male adolescents would initiate sexual activity early compared to their female counterparts.

## METHODS

### Participants

The participants in this study were part of "The Hanover Teen Study" conducted to identify factors associated with adolescent sexual activity in Jamaica[17]. Briefly the study was a cross-sectional survey of students attending different types of public high schools in the rural parish of Hanover, Jamaica. Schools in the parish were grouped based on method of admission. The schools selected for participation in this study were comprehensive high schools formerly new secondary schools (group 1), and technical and agricultural high schools (group 2). Students who perform well in the National Assessment Examination taken at the end of elementary schools attend academically rigorous secondary high school and technical high schools, which prepare them for college. On the other hand, admission to new secondary (now comprehensive high schools) providing a mixture of academic and vocational training is gained from feeder schools and those who did not perform well on the National Assessment Examination with little chance of continuing their education beyond the secondary level[2]. Seven hundred and eighty eight students attending the selected schools participated in the survey. For the purposes of this study, the 469 participants who indicated they had previously engaged in sexual intercourse were included in the analysis. However, we also defined composite scores for this study by taking sums of measures. The internal consistencies of these measures were assessed using Cronbach's alpha[18] based on data available for the original 788 participants.

### Procedure

The protocol for this study was reviewed and approved by the institutional review board (IRB) of the University of Alabama at Birmingham and the Ethics Committee, Ministry of Health, Jamaica. Written informed consent from parents and the students were obtained. Questions were read to the students to account for low literacy among some students, which was detected during pre-testing. To guarantee confidentiality, students did not put their name on the questionnaires and the investigators, and not the teachers, administered and collected the questionnaires. The procedure used for data collection and quality control has been described previously[17].

### Instruments and Measures

The survey instrument for this study was developed based on a comprehensive review of the literature on factors associated with adolescent involvement in sexual activity. The self-report questionnaire included questions on socio-demographic, individual, and family context variables.

### *Socio-Demographics*

Socio-demographic data was collected on variables such as current age, gender, type of school attended, and adolescent leisure (after school) activities. The type of school attended in this study was defined as comprehensive versus other high schools. Questions on leisure activities such as going straight home after school, hanging out with classmates, hanging out with girlfriend/boyfriend were answered “yes or no”. Adolescents were asked to indicate the characteristic of their first sexual partner. Response options included boyfriend/girlfriend, just a friend, a stranger, and relatives. This variable was dichotomized as boyfriend/girlfriend versus others.

### *Family Context Variables*

Family structure was measured by whether the adolescent lived in an intact two-parent family or not. We defined intact family as married biological parents living in the same household with the adolescent.

Parental monitoring and supervision of adolescent activities was measured using three items to determine the extent to which parents know their child’s whereabouts, how much interest they show in who they spend time with, and know what they do during their free time. An example of this item is “My parents know where I go after school and weekends”. Response options range from 1 (all the time) to 5 (none of the time). A composite variable based on the sum of these three items was constructed to determine the level of monitoring of adolescents’ activities by the parents. A high score on this scale is considered as lack of parental supervision/monitoring (Cronbach’s alpha = 0.54)

Perception of parental affection and support was a four-item measure (each for the mother and the father). An example would be: “I feel loved by my mother/father.” Response options to these questions range from 1 (all the time) to 4 (none of the time). Adolescents who were not living with their biological parents were asked to substitute “primary female or male guardian” for “mother/father”. Scores were combined separately for each parent to evaluate the level of affection between the adolescent and each of their biological or surrogate parents. High scores indicate lack of maternal affection and support (Cronbach’s alpha = 0.74) and lack of paternal affection and support (Cronbach’s alpha = 0.83).

### *Psychosocial Variables*

Adolescents’ norms towards abstaining from sex were assessed with five items. A sample question is “Do you think teenagers should wait until they are older than 18 years to start having sex?” Response options range from 1 (yes) to 3 (no). A composite variable based on the sum of scores on these items was constructed to determine adolescents’ attitudes towards premature sex. High scores indicate permissive sex norms (Cronbach’s alpha = 0.80).

Norms about negative sexual outcomes was a two-item scale that assessed adolescents’ attitude towards teenage pregnancy (e.g., “Do you think you would feel really badly if you get pregnant or if you get someone pregnant as a school boy”). Response options range from 1 (yes) to 3 (no). A combined variable based on the sum of scores on these two items was constructed to determine adolescents’ feelings towards negative sexual outcomes. A high score is interpreted as liberal attitude towards negative sexual outcomes and indicates adolescents’ lack of worries about getting pregnant or making somebody pregnant (Cronbach’s alpha = 0.59). The sex norms and negative sexual outcomes scales are based on the Alabama Rural Youth Survey instrument with reported reliability of 0.69 to 0.78[19].

We used a short form of the Beck’s Depression inventory II (BDI II) to measure depressive symptoms[20]. The depressive symptom measure consisted of three-items (sadness, tiredness, and suicidal thoughts). BDI II measures the severity of self-reported depression in adolescents and adults. The three symptoms were selected based on their high loadings on factor analysis and ease of response by adolescents. Respondents were asked to describe themselves in relation to these symptoms in the past two weeks including the day of the interview. Each symptom is rated on a 4-point scale ranging from 0-3 (Cronbach's alpha = 0.79). A high score is defined as reporting depressive symptoms.

### *Outcome Variable*

For this study, the outcome of interest is whether first sexual intercourse is experienced early or late. We defined early sexual debut as initiation of sexual intercourse before age 16 and late sexual debut as initiation of sexual intercourse at age 16 and above. We obtained this information from the response of adolescents to the question "Have you ever had sex?" (Yes/No). This question was followed by: If yes, how old were you the first time you had sex (age in years)? In this study, sex was defined as a penis entering the vagina. Given the sensitive nature of questions related to sexual behavior, these questions were asked towards the end of the survey after questions about adolescents' personal and family characteristics had been answered.

### Statistical Analysis

Frequency distributions were computed for selected socio-demographic characteristics. Mean values and standard errors of the mean (SEM) were calculated for continuous variables. We used Pearson's chi-square and t-tests to assess the statistical significance of the differences of these characteristics and age at sexual debut. We then performed logistic regressions between the outcome variable and each of the explanatory variables (bivariate) and between the outcome variable and all explanatory variables in the model (multivariate) to estimate crude and adjusted odds ratio (OR) with 95% confidence intervals (CI). Results were presented as unadjusted and adjusted ORs with 95% CI, to enable the assessment of the stability of the association when all covariates were adjusted for in the model. The final prediction model for early versus late sexual debut was reported. In the final model, variables were eliminated using stepwise and backward strategy with a 10% cut-off margin. Variables in the final model were fitted for each gender, to assess effect modification. All tests were two-tailed, and a p-value of 0.05 or less was considered as statistically significant. The Nagelkerke's R-Square statistic which measures how much variation in the dependent variable the model accounts for was used in this study to assess the strength of the association predicted by the final model (21). Statistical analysis was conducted using the Statistical Package for the Social Sciences (SPSS 14; SPSS Inc., Chicago, Illinois).

## RESULTS

Of the 469 sexually active adolescents, 45% (n= 211) were males and 55% (n= 258) were females. The mean age at initiation of sexual intercourse for males and females was 11 and 15 years respectively. The results of the comparison tests for the variables according to age at sexual debut are presented in Table 1. Overall, girls were less likely to initiate sexual intercourse early compared to boys ($p<0.0001$). Early starters were younger (at the time of the survey) than the late starters ($p<0.0001$), mean age 16 and 17 years respectively. They were more likely to report first intercourse with persons other than steady boyfriends or girlfriends ($p<0.0001$), attended comprehensive high schools ($p<0.0001$), and reported permissive norms about negative sexual outcomes ($p<0.0001$) and lack of parental monitoring ($p=0.003$). In comparison to late starters, early starters were less likely to go straight home after school ($p=0.003$) and less likely to live with their biological parents ($p=0.02$).

The ORs for age, gender, and permissive norms about negative sexual outcomes as a predictor of early sexual debut remained stable and significant after adjusting for all individual and family level variables. The unadjusted OR for relationship with partner at first intercourse was significant but with wide CIs, accounting for the small sample of late debutants initiating sex with partners other than steady boyfriend or girlfriend. After adjusting for other covariates, the OR and CI declined but remained statistically significant. The OR between parental monitoring, maternal affection and support, and paternal affection and support, and early sexual debut remained stable after adjusting for other covariates, but did not support significant association with early sexual debut. The effects of family structure, going home after school, and the type of school attended diminished and were no longer significant in multivariate analysis (Table 2).

**TABLE 1**

**Comparison of socio-demographic and family characteristics of participants by age at first intercourse**

| Characteristic, N (%), mean ± SEM[1] | All N= 469 | Age at first intercourse <16 years (N=367) | ≥16 years (N=102) | p-value |
|---|---|---|---|---|
| ***Socio-demographic*** | | | | |
| Age at time of survey | 16.5 ± 0.1 | 16.2 ± 0.1 | 17.4 ± 0.1 | <0.0001 |
| Female | 258 (55.0) | 165 (45.0) | 93 (91.2) | <0.0001 |
| First sexual partner (not a steady boyfriend/girlfriend) | 164 (35.0) | 157 (42.8) | 7 (6.9) | <0.0001 |
| Attending comprehensive high school | 321 (68.4) | 275 (74.9) | 46 (45.1) | <0.0001 |
| Goes straight home after school | 335 (71.4) | 250 (68.1) | 85 (83.3) | 0.003 |
| Hangs out with classmates | 179 (38.2) | 142 (38.7) | 37 (36.3) | 0.66 |
| Hangs out with boyfriend or girlfriend | 139 (29.6) | 107 (29.2) | 32 (31.4) | 0.66 |
| ***Family characteristics*** | | | | |
| Parental monitoring[2] | 6.0 ± 0.1 | 6.1 ± 0.1 | 5.8 ± 0.2 | 0.18 |
| Living with both parents | 169 (36.0) | 117 (31.9) | 52 (51.0) | 0.02 |
| Maternal affection and support[3] | 7.6 ± 0.1 | 7.5 ± 0.2 | 7.8 ± 0.3 | 0.29 |
| Paternal affection and support[4] | 10.3 ± 0.2 | 11.2 ± 0.4 | 10.0 ± 0.2 | 0.003 |
| ***Psychosocial variables*** | | | | |
| Depressive symptoms[5] | 3.0 ± 0.1 | 2.9 ± 0.1 | 3.0 ± 0.1 | 0.61 |
| Norms about refraining from sex[6] | 7.9 ± 0.1 | 8.0 ± 0.1 | 7.7 ± 0.2 | 0.10 |
| Norms about negative sexual outcomes[7] | 3.0 ± 0.0 | 3.1 ± 0.1 | 2.6 ± 0.1 | <0.0001 |

[1]Standard error of the mean

[2]A composite score representing parental monitoring (higher score represent lack of parental monitoring)

[3]A composite score representing maternal affection and support (higher score represent lack of maternal affection and support)

[4]A composite score representing paternal affection and support (higher score represent lack of paternal affection and support)

[5]A composite score representing depressive symptoms (higher score represent reporting depressive symptoms)

[6]A composite score representing norms about refraining from sex (higher score represent permissive sex norms)

[7]A composite score representing norms about negative sexual outcomes (higher score represent liberal attitude towards negative sexual outcomes e.g. pregnancy)

**TABLE 2**
**Unadjusted and adjusted estimates of association of individual and family characteristics with early age at first sexual intercourse**

| | Unadjusted (N=469) | | | Adjusted[3] (N=469) | | |
|---|---|---|---|---|---|---|
| Characteristic | OR[1] | 95% CI[2] | p-value | OR | 95% CI | p-value |
| Age at time of survey | 0.4 | 0.36-0.53 | <0.0001 | 0.4 | 0.29-0.56 | <0.0001 |
| Female vs. male | 0.1 | 0.04-0.16 | <0.0001 | 0.2 | 0.08-0.44 | <0.0001 |
| First sexual partner (not a steady boyfriend/girlfriend) | 10.1 | 4.58-22.47 | <0.0001 | 4.7 | 1.74-12.62 | 0.002 |
| Goes straight home after school | 0.4 | 0.24-0.75 | 0.003 | 0.7 | 0.34-1.46 | 0.34 |
| Hangs out with classmates | 1.1 | 0.70-1.75 | 0.66 | 0.9 | 0.47-1.65 | 0.69 |
| Hangs out with boyfriends/girlfriends | 0.9 | 0.56-1.45 | 0.66 | 1.4 | 0.70-2.68 | 0.35 |
| Comprehensive high school vs. others | 3.6 | 2.31-5.74 | <0.0001 | 1.2 | 0.53-2.63 | 0.69 |
| Parental monitoring | 1.1 | 0.97-1.21 | 0.18 | 1.1 | 0.94-1.32 | 0.22 |
| Living with both parents | 0.6 | 0.35-0.93 | 0.02 | 1.0 | 0.52-1.77 | 0.90 |
| Maternal affection and support | 1.0 | 0.89-1.04 | 0.29 | 1.0 | 0.90-1.14 | 0.84 |
| Paternal affection and support | 1.1 | 1.03-1.17 | 0.003 | 1.1 | 0.97-1.17 | 0.16 |
| Norms about refraining from sex | 1.1 | 0.98-1.26 | 0.10 | 0.9 | 0.78-1.07 | 0.29 |
| Attitude towards negative sexual outcomes | 2.0 | 1.45-2.60 | <0.0001 | 2.0 | 1.33-2.91 | 0.001 |
| Depressive symptoms | 1.0 | 0.84-1.11 | 0.61 | 1.0 | 0.80-1.22 | 0. 92 |

[1]OR: Odds ratio [2]CI: Confidence Interval. [3]Adjusted for all characteristics listed.

The result of the final predictive model is presented in Table 3. All variables that were significant at the multivariate level remain significant in the overall final model with some effect modification by gender. Female adolescents who engaged in sexual activity early were more likely to have been initiated by a partner other than a steady boyfriend (OR= 11.95, 95% CI= 2.39-59.69), and had permissive norms about negative sexual outcomes (OR= 1.83, 95% CI= 1.21-2.77). Males were mainly influenced by permissive norms towards negative sexual outcomes (OR= 3.11, 95% CI= 1.09-8.93). Being older at the time of survey appears to be protective for both genders. The overall model has a predictive efficacy of 48%.

**TABLE 3**
**Final model predicting factors associated with early sexual intercourse stratified by gender**

| Characteristic | Overall (N=469) | | | Male (N=102) | | | Female (N=367) | | |
|---|---|---|---|---|---|---|---|---|---|
| | OR[a] | 95% CI[b] | p-value | OR | 95% CI | p-value | OR | 95% CI | p-value |
| Female | 0.16 | 0.07-0.36 | <0.0001 | | | | | | |
| Age at time of survey | 0.40 | 0.32-0.52 | <0.0001 | 0.42 | 0.23-0.77 | 0.005 | 0.39 | 0.30-0.52 | <0.0001 |
| First sexual partner (not a steady boyfriend/girlfriend) | 4.19 | 1.62-10.84 | 0.003 | 1.28 | 0.31-5.32 | 0.74 | 11.95 | 2.39-59.69 | 0.003 |
| Norms about negative sexual outcomes | 1.96 | 1.34-2.87 | 0.001 | 3.11 | 1.09-8.93 | 0.04 | 1.83 | 1.21-2.77 | 0.004 |
| Model statistics | | | | | | | | | |
| Nagelkerke $R^2$ | | 0.48* | | | 0.20* | | | 0.37* | |

[a]OR: Odds ratio. [b]CI: Confidence Interval. * R2 change is based on Nagelkerke's statistics

## DISCUSSION

We investigated factors associated with initiation of sexual intercourse before the age of 16 among rural Jamaican adolescents, based on an increasing concern regarding negative consequences of early sexual debut in this population. Our findings indicate that early sexual experience is common. The mean age at first sexual intercourse was 11 years for boys and 15 years for girls. This is in agreement with findings from previous adolescent surveys in the country[2,6]. These findings have implications for prevention of adolescent pregnancy and STIs - programs that attempt to delay age at first sex need to start early in primary school, before young people reach puberty or become sexually active.

An interesting finding is the association of type of first sexual partner and early sexual debut among female adolescents. Girls whose first sexual partner was not a steady boyfriend were more likely to be early starters. Type of early sexual partners reported in focus group discussions (FGDs) by Jamaican female adolescents included family members, usually the father, stepfather, cousins, uncles and brothers[14]. Other older men in the community were also mentioned, suggesting that such relationships could be coercive or transactional. According to the Jamaican Injury Surveillance System, 86% of sexual assault cases in 2002 and 2003 were committed by a relative, a friend, an acquaintance or an intimate partner[22]. We cannot determine the extent to which sexual intercourse with people other than steady partners represent coerced or transactional sexual relationships from our data. Further investigation is required to fully understand this association.

Another important finding, similar to reports from previous qualitative studies is the liberal attitude of some Jamaican adolescents towards negative sexual outcomes, such as pregnancy. In our study, male and female adolescents with permissive norms about negative sexual outcomes reported early coital debut, the association however was stronger for boys than for girls. Jackson et al., in their report of FGDs conducted among Jamaican high school students reported that boys were more likely than girls to express enthusiasm about an unexpected pregnancy[8]. Male participants in the discussion reported that a boy who impregnated a girl is often the object of his ‘friends’ admiration and envy. Similar observations were reported by Kempadoo and Dunn[15]. Girls bear the consequences of adolescent pregnancy more than boys; this could explain why they are not as enthusiastic by the prospect of being pregnant as boys. In Jamaica, culture and gender norms impose different standards on males and females. Males have been reported to have a macho attitude to sexuality and are egoistic about their sexual exploits[8]. Conservative sexual ideals, coexisting with tacit acceptance of early pregnancy in Jamaica, may promote this attitude towards adolescent pregnancy[23]. It is probable that many adolescents might not have sex, if some norms concerning sexual behavior can be changed; however, the effectiveness of any strategy in changing norms will depend on whether there is agreement across age groups in society about the appropriate context for sexual behavior[24].

Several of our hypothesized measures failed to distinguish between early and late initiators. The relationship found in some studies between family structure and early sexual intercourse was not obvious here[14]. While it could be argued that family structure, particularly living in two-parent households could be related to whether or not one abstain from sex, in this study it did not influence the timing of first sexual experience. It is possible that we were unable to detect the association due to the small sample size of those who were debuting after age 16 and did not live with both parents. It may also suggest that early sexual debut is strongly determined by other factors (e.g. relationship with first sexual partner and attitude towards sexual outcomes) and that living in two parent families does not have as much influence. Several other studies have also reported lack of association between family structure and early sexual debut[24]. One potential explanation of the lack of association between family structure and age of sexual debut is that the family environment of early starters may actually not be protective. Young adolescents, especially females could have been initiated into sex by relatives or acquaintances[15]. Similarly, we did not find any association between parental monitoring and early sexual debut.

The non-significance of an association between early sexual initiation and leisure activities, such as, hanging out with boyfriends, or classmates is notable. It again reinforced our finding that first sexual intercourse in early adolescence might not occur in a socio-romantic setting, especially for girls. Early sexual initiation may likely be coercive rather than romantic[15].

Against popular typecast; the type of school attended did not independently affect the timing of sexual initiation. It has been suggested that adolescents attending comprehensive high schools (formerly new secondary schools) are more likely to initiate sexual intercourse early because of the limited potential for further education associated with these schools[2]. Students attending these schools had been reported to suffer from low self esteem[25], a trait that may be related to early sexual activity. This association, which was significant at the bivariate level, was insignificant in the multivariate analysis, indicating that some other characteristics of adolescents attending these schools may be different.

## Limitations, Implications, and Future Directions

Limitations in the study design and data collection required that findings should be interpreted with caution. We relied on self-reports of sexual activity. Due to the sensitive nature of some of the questions, some adolescents may give socially desirable answers. This tendency however, may have been minimized by the anonymity entrenched in data collection. Insufficient power or small cell samples could also have affected some of our findings. Finally, the cross-sectional design of this study may be a major limitation as only association, but not causal inferences can be made.

Our study was a unique opportunity to study sexual behavior of rural adolescents in Jamaica, who have traditionally been underrepresented in previous studies. While the findings may not be representative of all adolescents from different socio-economic and demographic backgrounds in the country, the findings provide insight into the sexual behavior of adolescents within a sub-set of the Jamaican society. A major strength is the assessment of individual and family variables at the same time, thereby reducing the number of unmeasured covariates that could bias our findings. However, our multivariate model accounted for 48% of the variations in age at first intercourse, so we can not assume that the factors examined here are the only ones affecting early initiation of sexual intercourse.

This research shows that individual factors (with slight modification for males and females), determine initiation of sexual intercourse before the age of 16 in Jamaica. None of the family level variables were independent risk or protective factors. Most sexually experienced adolescents in rural Jamaica engaged in their first sexual intercourse between the ages of 11 and 15, with males initiating sexual intercourse earlier than females. To maximize their preventive benefits, programs that attempt to delay age at first sex should begin early in primary school before adolescents become sexually active.

A major concern from our findings is that young female adolescents who engaged in sexual activity early were initiated by partners who were not steady boyfriends. The extent to which this represents incestuous and/or coercive sexual relations warrants further investigation. Considering the high rates of HIV and adolescent pregnancy in this population, reproductive health programs are needed to meet the demand for information and services among these high-risk youth.

## ACKNOWLEDGEMENTS

This work was partly supported by the Minority International Research Training Grant (#T37-TW00077) awarded to the last author (Dr. Pauline Jolly) by Fogarty International Center, National Institute of Health, and the Ministry of Health, Jamaica. The authors of this article take responsibility for its contents and not the National Institutes of Health or the Ministry of Health, Jamaica. The authors would like to thank the principals and students of the schools in the parish of Hanover, Jamaica who participated in the study.
